# Intentional maintenance of antiphase bimanual pattern at transition frequency: Is it associated with inhibition processes?

**DOI:** 10.1016/j.heliyon.2023.e16089

**Published:** 2023-05-09

**Authors:** Rita Sleimen-Malkoun, Louise Devillers-Réolon, Jean-Jacques Temprado

**Affiliations:** Aix-Marseille University, CNRS, ISM, 163 av. de Luminy, 13288 Marseille cedex 09, France

**Keywords:** Bimanual coordination, Intentional dynamics, Movement transition, Perceptual inhibition, Motor inhibition

## Abstract

This study aimed at demonstrating the intentional modulation of bimanual coordination dynamics at transition frequency and determining whether it is associated with perceptual and/or motor inhibition capacities. Healthy adults (N = 29) performed in a random order: i) bimanual anti-phase (AP) movements at the maximal individual transition frequency, with the instruction to either let go, or intentionally maintain the initial movement pattern and oppose to the spontaneous transition to in-phase (IP) movements, and ii) The Motor and Perceptual Inhibition Test, giving separate scores for perceptual and motor inhibition. Results showed that in the intentional condition participants were able to delay (more movement cycles before the transition) and suppress (more trials without transition) the spontaneous transition from AP to IP. A statistically significant, though weak, correlation was found between motor performance and perceptual inhibition scores. We interpreted our findings as an indicator of the presence of an inhibitory mechanism underlying intentional dynamics that is partially associated to perceptual inhibition in healthy adults. This could have implications in populations with compromised inhibitory capacities, which might entail motor repercussions, and suggests the possibility of using bimanual coordination as means to stimulate both cognitive and motor capacities.

## Highlights

• Intentional modulation of spontaneous dynamics is possible at transition frequency.
• Healthy adults can intentionally delay and suppress the spontaneous transition.
• Intentional motor performance positively correlates with perceptual inhibition score.

## 1 Introduction

Coordinated bimanual movements are at the center of many daily actions and activities. Their stability and flexibility result from a coalition of multiple constraints, that are of musculoskeletal, perceptual, cognitive, and neural origins [[1], [2], [3]]. In this context, the interplay between cognitive and (sensori)motor processes is particularly important to understand, as it is the main source of behavioral adaptability of bimanual patterns to task demands.

Cyclical bimanual coordination has been a widely used paradigm for studying cognitive-motor interactions underlying complex movements (e.g., Refs. [[4], [5], [6], [7]]). Indeed, it allows distinguishing between intrinsic and intentional dynamics of coordination patterns. Intrinsic dynamics is characterized by the existence of two spontaneously stable movement patterns, and the transition from one to another when movement frequency is progressively increased [8]. Specifically, the two identified patterns are the in-phase (IP) pattern, resulting from the simultaneous activation of homologous muscles and giving rise to mirror symmetrical movement with respect to the body midline, and the anti-phase (AP) pattern, resulting from the alternate activation of homologous muscles and giving rise to parallel movement. Coordination patterns can be characterized by computing the relative phase (RP) that quantifies the spatiotemporal relationship between the limbs [9]. Accordingly, the IP coordination pattern is characterized by 0° RP values, and the AP pattern by 180° RP values. The IP pattern has been shown to be intrinsically more stable than the AP pattern [10], with a spontaneous transition occurring from AP to IP when movement frequency increases beyond a critical threshold [8,11], named transition frequency.

To meet task demands, the intrinsic dynamics can be modulated by cognition [12]. Specifically, previous studies suggest that cognition (e.g., attention and inhibition) involvement can delay or inhibit the spontaneous transition from AP to IP [6,13]. Attention has been the most studied cognitive function in this regard [5,14], with attentional investment presumably increasing coupling strength between the two oscillating hands. Nevertheless, a previous study by Temprado et al. [6], on the effects of learning on bimanual patterns stabilization capacities, showed that, following training, participants were able to maintain a higher level of stability without necessarily decreasing the associated attentional cost. This finding suggests that a different mechanism than attention could be also involved in this gain of stability during training. A candidate mechanism could be inhibition that might also be involved in intentional stabilization of the AP pattern by precluding the emergence of the IP pattern as a spontaneous response at high oscillation frequency. Recently, through correlations with Stroop task [15] performance, Temprado et al. [4] suggested the involvement of inhibition in maintaining the AP pattern and intentionally switching to IP (see Ref. [16] for a converging conclusion based on neural evidence). Converging results were found in a recently submitted study on the effects of training on intentional switching and the inhibition of the AP to IP transition in elderly [42]. Though plausible, the involvement of inhibition when seeking to intentionally maintain the less stable AP pattern and resist the spontaneous transition to the IP pattern remains to be demonstrated. We argue that this inhibitory mechanism would be reflected through the increase in the number of movement trials without transition and the number of movement cycles before the transition. It might additionally be dependent on the cognitive inhibition capacity of the individuals.

Inhibition is unarguably a multifaceted process that can be studied through different paradigms, and has been accounted for from different theoretical perspectives. For instance, the Stroop task [15] has been classically used to assess cognitive inhibition capacities, in an undifferentiated way [17]. In cognitive psychology, the presence of different components of inhibition has been discussed in the Dual Mechanism of Control Framework distinguishing between proactive and reactive inhibitory control [[18], [19], [20]]. The results reported by Maslovat et al. [21] suggest that this distinction is heuristic in the context of intentional switching triggered by an auditory signal. In a different framework, inhibition has been characterized through two overlapped, but separable, processes: perceptual and motor inhibition, [[22], [23], [24]]. Since bimanual coordination dynamics have been shown to be governed by both perceptual (e.g., visual perception of inter-limb phase relationship) and motor constraints (e.g., recruitment of homologous or non-homologous muscles) [[25], [26], [27], [28]], we consider it relevant to distinguish the potential roles that could be played, separately, by perceptual and motor inhibition. While perceptual inhibition plays a role in preventing interference from task-irrelevant stimuli [22,24,29], motor inhibition enables the suppression of prevalent but inappropriate motor responses [22,24,29,30]. Perceptual inhibition has been shown to contribute more specifically to sensory integration in motor control through the study of postural control, which relays on continuous control processes, as those involved in cyclical bimanual coordination [29,31]. Accordingly, in bimanual coordination, we contend that perceptual inhibition might contribute to channelling the participants' attentional resources towards task-relevant perceptual information (visual, auditory, and/or proprioceptive). Thereby, it would reinforce the capacity to bring back the behavior to the initial AP pattern (considered as the perceptual goal) whenever it tends to escape towards the more comfortable IP pattern (considered as the distractor).

One way to evaluate perceptual and motor inhibition capacities through separate scores is to use the Motor and Perceptual Inhibition Test (MAPIT) [22,32]. The MAPIT has been shown to be relevant to investigate at least the perceptual involvement of inhibition in the control of complex movements [29,31,33,34]. Although motor inhibition as measured by the MAPIT hasn’t been shown so far to correlate with motor performance, it might still be involved in suppressing the onset of the automatic transition to IP that represents the default inter-limb coupling mode at high movement frequency.

Overall, in this study, we were interested in demonstrating the intentional modulation of bimanual dynamics at transition frequency and determining whether it is associated with perceptual and/or motor inhibition capacities. Participants performed, in a conter-balanced order, the MAPIT, and bimanual AP movements at transition frequency with the instruction to either let go or intentionally maintain AP and oppose to the spontaneous transition to IP. To analyze motor performance, we compared the number of trials without transition and the number of movement cycles before the transition between the two instruction conditions. We were expecting to observe more trials without transition and more movement cycles before the transition in the intentional maintenance condition. Regression analyses were then conducted between inhibition scores and motor performance change from the “let go” to the “maintain and resist” condition. We were expecting both perceptual and motor inhibition capacities to correlate with AP pattern intentional maintenance, with potentially a stronger correlation with perceptual inhibition.

## 2 Method

### 2.1 Participants

Twenty-nine healthy volunteers (32.35 ± 11.63 years, 17 women) participated in this study. None of the participants self-declared having previous or current neurological or psychiatric disorders, recent or current musculoskeletal upper limb problems, an ongoing psychopharmacological medication, an uncorrected vision, an addiction to illegal substances or alcoholism. Following self-report of their hand use, 26 of our participants were right-handed, and only three were left-handed.

Prior to their enrolment, all participants were given detailed written information about the study, without stating its precise objective or the underlying hypotheses. They all gave their written informed consent to the experimental procedure that agreed with the Declaration of Helsinki and was approved by the Ethics Committee for Research in Science and Techniques of Physical and Sports Activities (CER STAPS n° IRB00012476-2020-15-12-78).

### 2.2 Tasks and apparatus

Participants performed the bimanual and inhibition tasks presented below in a randomized counter-balanced order. During all the experimental session, they were seated in a height adjustable chair without armrests, with their back straight leaning against the seatback.

#### 2.2.1 The bimanual coordination task

The experimental setup was the same as the one used in Temprado et al. (2010, 2020) [4,11]. It consisted of pronation-supination movements of the forearms in the frontal plane using rotating handles with elbows flexed at 90°. Handles movements were recorded at a sampling frequency of 100 Hz using two potentiometers placed on the axis of rotation of each handle. Participants were instructed to produce the required bimanual movement pattern as accurately and continuously as possible, while preserving a large amplitude (at least 45° around the central position), and in synchrony with an auditory metronome prescribing the required frequency through a speaker (Beats Pill 2.0 by Dr Dre, Santa Monica, USA). A full movement cycle had to be performed between two subsequent metronome beats.

The task began with a familiarization period, during which participants performed 50 cycles of IP and AP bimanual movements at 1 Hz and at 1.5 Hz. Then, participants performed a block of 6 trials to identify their transition frequency, that is the frequency for which a spontaneous transition from AP to IP occurred. During these 6 trials, the metronome frequency was increased stepwise by 0.5 Hz every 10 s, starting from 1 Hz until the occurrence of the transition. Participants were instructed to adopt the AP pattern, and not to intervene (let go) if they felt the need at some point to switch to the IP pattern. The transition frequency was defined as the higher frequency of the 6 trials at which the participants switched from AP to IP. The individual transition frequency was used to perform the experimental task under two conditions presented in a randomized order: the “let go” condition [7,8] to observe the spontaneous dynamic, and the “maintain and resist” condition [6,13] to observe the intentional dynamics. In the “let go” condition, as for the transition frequency identification phase, participants were instructed to adopt the AP pattern and not to intervene or resist if transition to IP tended to occur naturally. In the “maintain and resist” condition, participants were instructed to maintain the AP pattern as long as possible and resist the spontaneous tendency to switch to the IP pattern. In both conditions, if a transition occurred, participants were asked to continue until the end of the trial with the new adopted IP pattern and not to attempt to go back to the initial AP pattern. Participants performed 6 trials of 50 movement cycles in both conditions.

For each trial, we calculated the RP (in degrees) between the oscillations of the right and left hands, with the right hand as reference. The RP represented the difference in phase angles between the two hands:

$$RP=\frac{t1-t2}{T}\times 360{}^{\circ}$$

with t1 being the time in seconds of the right hand’s peak (maximal or minimal), t2 the time in seconds of the left hand’s peak, and T the period in seconds of the right hand. The maintenance of the AP pattern was assessed using two complementary indicators: (i) the number of trials without transition (NT) and, (ii) the number of cycles before transition (NC). The onset of the transition was set as the first value of three consecutive cycles of the RP under 135°. If there was a transition, RP was calculated only before the transition onset. If there was no transition, NC was the total number of cycles of the corresponding trial. Higher NT and NC indicated better capacities to maintain the AP pattern and resist the spontaneous transition from AP to IP.

#### 2.2.2 The Motor and Perceptual Inhibition Test (MAPIT)

Motor and perceptual inhibition capacities were assessed using the Motor and Perceptual Inhibition Test (MAPIT) [22], that was validated by Nassauer and Halperin (2003) [22]. The test interface was computerized using ICE ® programming software (https://trello.com/b/EtNCNrZH/ice). The MAPIT is a reaction time task that requires responding by pressing as fast as possible a left or right key according to the direction (left or right) or the location (left, center or right) of a black arrow presented on a white background computer screen (here a Dell24 P2418HT, 23.8 inches). In this study, the participant responded using a modified AZERTY keyboard on which only two letter keys were kept “Q” (corresponding to the answer “left”) and “M” (corresponding to the answer “right”). Participants were seated comfortably in front of the setup holding lightly without pressing their right index on the “M” key and their left index on the “Q” key. As soon as the stimuli appeared on the screen, they were requested to press as fast as possible the adequate key according to the given instruction before each set of the test. In between sets, a central fixation cross was presented on the screen, and disappeared with the onset of the stimulus. The presented stimulus remained on the screen until the participant responded.

**Fig. 1:**
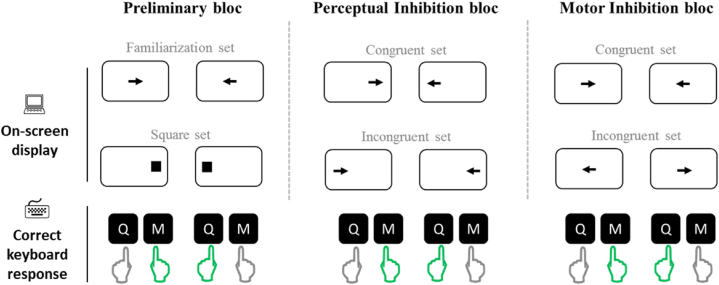
Illustration of the MAPIT. The on-screen display and the expected correct response are presented for all the 12 conditions of the test. Each of the three blocks (Preliminary, Perceptual inhibition, and Motor inhibition) included two sets with two randomized conditions. Only the sets of the perceptual inhibition block were intermixed. The correct keyboard letter to press (Q with left index or M with right index) as soon as the on-screen stimulus is presented is indicated by the green hand on the last row. For sake of simplicity, in the different blocks, we depicted the conditions requiring the same response in the same column. (For interpretation of the references to colour in this figure legend, the reader is referred to the Web version of this article.)

The test included three blocks of 80 trials each presented in a fixed order (Fig. 1): a preliminary block, a perceptual inhibition block, and a motor inhibition block.

The preliminary block included two sets. The first was a familiarization set. It consisted of two conditions, one with 20 arrows pointing left and one with 20 arrows pointing right. The left and right pointing arrows were presented in a random order at the center of the screen. The participant was required to press the key corresponding to the direction pointed by the arrow (e.g., pressing Q with the left finger when the arrow points to the left). The second set was used to elicit and reinforce the prepotent spatial responses. It consisted of two conditions, one with 20 black squares presented on left side of the screen (8.5 cm from the center), and one with 20 black squares presented on the right side of the screen (8.5 cm from the center). The resulting 40 trials were presented randomly to the participant who needed to press the key according to the position of the square (e.g., press Q with the left finger when the square is presented on the left side of the screen).

The perceptual inhibition block included two combined sets (40 trials each, with 20 arrows in each direction). This resulted in 80 randomly presented trials. In the perceptual congruent set, the spatial location of the arrow corresponded to its pointing direction (e.g., left pointing arrow on the left side of the screen), while in the perceptual incongruent set the spatial location of the arrow conflicted with its pointing direction (e.g., left pointing arrow on the right side of the screen). Participants were required to press the key corresponding to the arrow direction regardless of its location on the screen. In the incongruent set, the participant had to inhibit the natural tendency of processing the arrow’s location, and instead to focus on its pointing direction.

The motor inhibition block included two separated sets (40 randomized trials each, with 20 arrows in each direction) wherein the arrows were always located at the center of the screen. In the motor congruent set, the participant had to respond in accordance with the arrows' direction. In the incongruent set, the participant had to respond the opposite to where the arrow pointed (e.g., pressing Q with the left finger when the arrow points to the right), inhibiting thereby the over-learned response that is spatially compatible with the presented stimulus.

For each condition of each set of every participant, the median response time in milliseconds (RT) of correct trials was calculated. We chose medians instead of means to minimize the influence of possible outliers. The perceptual inhibition (PI) and motor inhibition (MI) scores are derived from the median RTs as follows:

$$PI={Median\;RT}_{Perceptual\;incongrent}-{Median\;RT}_{Perceptual\;congruent}$$

$$MI={Median\;RT}_{Motor\;incongruent}-{Median\;RT}_{Motor\;congruent}$$

Lower MI and PI scores were respectively associated to higher motor inhibition and perceptual inhibition capacities [22,32].

### 2.3 Data processing and analysis

Data were processed with MATLAB R2018b (MathWorks, Natick, MA, USA) and Microsoft Excel 2010 (Microsoft Corporation, Impressa systems, Santa Rosa, California, USA). For each trial of the bimanual coordination task, we removed the first 3 s to ensure the stabilization of the participant’s behavior. Then raw data were filtered with a dual-path Butterworth filter (cut-off frequency 10 Hz, order 2), with application of the correction factor [35]. An amplitude centering procedure was used to remove possible frequency artifacts due to imperfect sinusoidal signals. If the transition occurred before the three first seconds that were removed and before having at least 8 values of RP (i.e., 4 cycles of coordination), the trial was discarded from RP and NC computation. These trials were only included in the dependent variable NT. They represented overall 5.17% of trials for the “let go” condition and 0% of trials for the “maintain and resist” condition. As participants performed the task in two different conditions, bimanual coordination data are reported as means with within-subject correlation-adjusted error bars (*M* ± *CI*), with CI representing the 95% confidence interval normalized to account for the within-subjects design [36,37].

For the MAPIT task, we ensured that all participants had more than 75% of correct answers and that none of the analyzed RT values exceeded 3 s or were below 200 ms. MAPIT data are reported as means with standard deviation (M ± SD).

All analyzed variable were checked for outliers outside the range of Mean ± 3 × SD. No outlier values were found.

### 2.4 Statistical analysis

The studied dependent variables were statistically analyzed using JASP software (version 0.14.1.0, JASP Team, Amsterdam, Netherlands). To verify the compliance with task instruction in the bimanual coordination task, we compared both conditions using a one-way repeated measure analysis of variance (ANOVA) with the factor condition (2: “let go”, “maintain and resist”). The level of significance was set to 5% (*p* < 0.05). Size effects are reported as partial eta square (η^2^). To determine whether and which inhibition processes could be associated with the ability to intentionally stabilize the AP bimanual coordination pattern and resist the spontaneous transition, simple linear regression analyses were conducted. In this analysis, the dependent variable was the participants change values from the “maintain and resist” condition to the “let go” condition (delta = “maintain and resist” value – “let go” value) for which a statistically significant effect of condition was found. The predictor was the MAPIT inhibition scores (MI, PI) analyzed separately. The coefficient of determination is reported as R^2^. For the sake of brevity, only values relative to statistically significant effects are reported in the results.

## 3 Results

### 3.1 Bimanual coordination performance

**Fig. 2:**
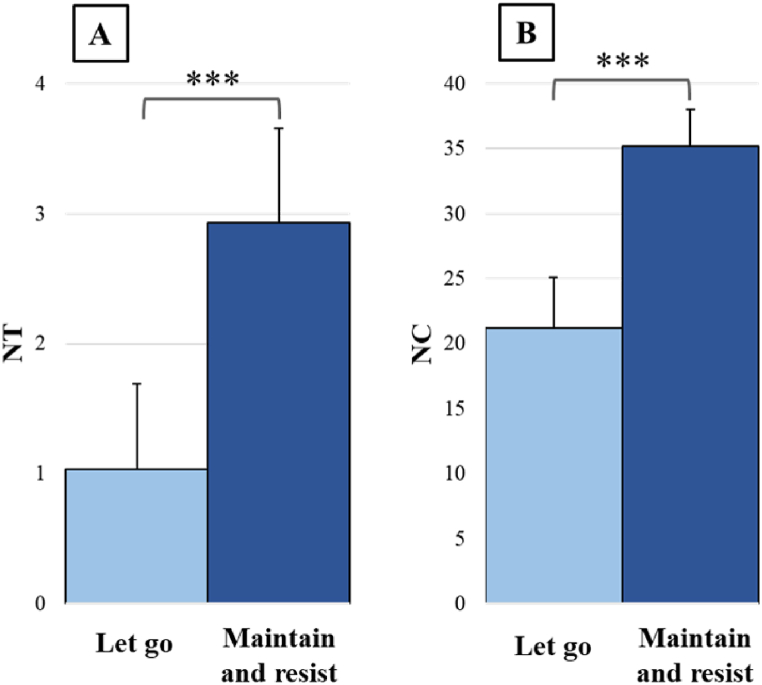
Bimanual coordination performance in the “let go” (light blue) and “maintain and resist” conditions (dark blue). (A) Number of trials without transition (NT), and (B) Number of cycles before transition (NC). Error bars represent the normalized 95% confidence interval. ***p ≤ 0.001. (For interpretation of the references to colour in this figure legend, the reader is referred to the Web version of this article.)

For the RP, the ANOVA revealed no significant effect of the condition factor, with a general mean around 180° representing the required AP pattern (“let go”: 161.71 ± 2.27°; “maintain and resist”: 160.59 ± 2.62°). The ANOVA also revealed a significant effect of condition for NT (F(1,28) = 46.59, *p* < 0.001, η^2^ = 0.625) and NC (F(1,28) = 60.27, *p* < 0.001, η^2^ = 0.683), with more trials without transition (Fig. 2A) and more cycles before the transition (Fig. 2B) in the “maintain and resist” condition (respectively, 2.93 ± 0.73 trials and 35.19 ± 2.85 cycles) than in the “let go” condition (respectively, 1.03 ± 0.66 trials and 21.21 ± 3.89 cycles).

### 3.2 Correlation between inhibition scores and bimanual performance

**Fig. 3:**
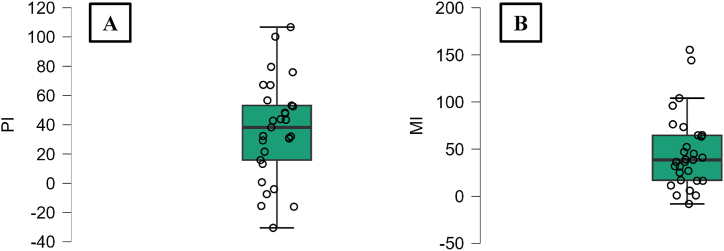
Boxplots for the MAPIT’s PI (A) and MI (B) scores.

**Fig. 4:**
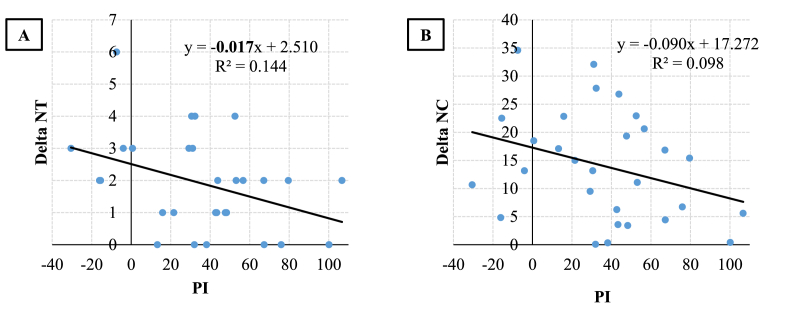
Linear regressions between perceptual inhibition scores (PI) and between-conditions delta of bimanual coordination variables. (A) Number of trials without transition (delta NT) and (B) Number of cycles before transition (delta NC). Significant results at 5% threshold are in bold.

The MAPIT results showed PI and MI scores of respectively 36.41 ± 33.69 ms and 46.68 ± 39.68 ms (Fig. 3). As the ANOVA revealed a significant effect of condition NT, and NC, we correlated the between-conditions delta of these two variables and the MAPIT inhibition scores (PI and MI). We only found statistically significant results for PI. A significant negative linear regression was found between delta NT and PI (F(1.27) = 4.55, *p* = 0.042, R^2^ = 0.144) (Fig. 4A), with delta NT decreasing by 0.017 trial for each millisecond increase of PI. This regression indicated that individuals having lower PI scores (better perceptual inhibition) achieved more trials without transition when attempting to intentionally stabilize their motor behavior. The linear regression between delta NC and PI was not statistically significant at the 5% threshold, but we observed a tendency towards a negative relation (F(1,27) = 2.942, *p* = 0.09, R^2^ = 0.09) (Fig. 4B). Participant’s delta NC tended to decrease by 0.09 cycle for each millisecond increase of PI, suggesting that individuals with lower PI scores tended to perform more cycles before transition in the “maintain and resist” condition.

## 4 Discussion

First of all, our results showed that healthy adults were able to intentionally delay and suppress the spontaneous transition from AP to IP when performing the task at their highest individual transition frequency. Also, we found that perceptual inhibition capacities, as measured by the MAPIT’s PI score, were to some extent associated with the observed intentional stabilization performance.

The capacity to intentionally maintain the AP pattern and delay or even suppress the spontaneous transition to IP at transition frequency was reflected by an increase in the number of movement cycles before transition, and an increase in the number of trials without transition in the “maintain and resist” condition, compared to the “let go” condition. These results were statistically robust with very large effect size (η^2^ = 0.62 for NT, and η^2^ = 0.7 for NC). They corroborate and extend the previous findings reported by Lee, Blandin and Proteau [13] who showed in a small sample that the spontaneous transition from AP to IP can be intentionally suppressed at different movement frequencies (1–3 Hz).

In the cognitive domain, our participant showed highly variable scores in the MAPIT. This appears to be inherent to the task itself, as reported in previous studies [29,31,33].

Nevertheless, as expected, the correlation analysis showed that higher perceptual inhibition capacities (lower PI scores) were to some extent associated with lower delta NT and tendentially with lower delta NC, that is with more trials without transition and more movement cycles before transition in the “maintain and resist” condition than in the “let go” condition. The effect sizes were however small, as attested by the regression’s slope and R^2^ values (0.017, and 0.14 respectively).

Overall, the observed bimanual behavior under the two task instructions together with the shown link between inhibitory and motor performances suggest the presence of an inhibitory mechanism contributing to the intentional maintenance of the AP pattern at transition frequency. Thus, it can be concluded that better perceptual inhibition may contribute to better intentional stabilization of the AP pattern at high movement frequency, through suppressing and delaying the spontaneous transition. The role that seems to be played by perceptual inhibition relative to motor inhibition gives support to the importance of perceptual constraints in bimanual coordination dynamics [25,26,38]. However, as aforementioned, our regression analysis showed a relatively low coefficient of determination. Hence, although the association between motor performance and perceptual inhibition was statistically significant, it was overall weak. This relation could be much stronger when considering older adults as previously reported in studies on postural control [29,31].

Motor constraints might also play a role, although a statistically significant link between MI and bimanual coordination performance was not found in this study. To be noted that this result remains unchanged even when removing the two extreme values noted in MI results (exceeding by 1.5 times the interquartile range, Fig. 3). Redfern et al. [31] suggested that motor inhibition could have a more specific contribution in situations where more active motor responses are required such as in response to perturbations. Thus, it could be more strongly involved when intentional switching between bimanual coordination patterns is required (Torre et al., in revision). Another possible explanation to this result could be that the motor inhibition process evaluated by the MAPIT is not the same as the one required in the intentional bimanual dynamics. Indeed, the MAPIT is a discrete perceptual-motor task, while the bimanual coordination paradigm is a continuous cyclic task belonging to a separate movement class [39]. It is noteworthy to acknowledge that motor inhibition processes involved in cancelling prepared discrete movements and stopping ongoing rhythmic movements are assumingly different [40,41].

Overall, although the MAPIT appears relevant to investigate at least the perceptual involvement of inhibition in the control of complex movements as successfully shown in other studies [29,31,33,34], the large variability of the obtained scores makes this test challenging to use and interpret.

## 5 Conclusions

The present study demonstrated how healthy adults were able to intentionally constrain their spontaneous coordination dynamics by delaying and suppressing the transition from AP to IP at their highest individual transition frequency. The capacity to intentionally maintain the initial AP pattern and resist the transition to IP at such high movement frequency appears to be to some extent linked to perceptual inhibition, but not to motor inhibition, at least as measured through the MAPIT’s PI score. We interpreted our findings as an indicator of the presence of two distinct, but not mutually exclusive, mechanisms underlying intentional dynamics: one relying on coupling enforcement of the ongoing behavior (observed mainly in dual-task studies, [6,14]), and the other (observed here) relying on the inhibition of the emergence of a new stable solution coordination pattern (see Refs. [16,26] for a consistent interpretation).

In future studies, inducing perceptual or motor conflicts could help better elucidate the contribution of these constraints of different nature that are long known to contribute in different ways to bimanual coordination [25,26,28]. Other perspectives of inhibitory control, like the Dual Mechanism of Control Framework [19] deserve to be investigated, as they might further clarify the inhibitory mechanisms involved in intentional bimanual stabilization. It would be also of interest to include other cognitive measures, notably of attention.

The observed results could have implications in special populations suffering from compromised inhibitory capacities, which might entail motor repercussions. It suggests the possibility of using the tested bimanual paradigm as means to stimulate both cognitive and motor capacities.

## Author contribution statement

Rita Sleimen-Malkoun, PhD, PT: Conceived and designed the experiments; Performed the experiments; Analyzed and interpreted the data; Wrote the paper.

Louise Devillers-Réolon, Msc: Performed the experiments; Analyzed and interpreted the data; Contributed reagents, materials, analysis tools or data; Wrote the paper.

Jean-Jacques Temprado, Prof: Conceived and designed the experiments; Wrote the paper.

## Data availability statement

Data will be made available on request.

## Declaration of competing interest

The authors declare that they have no known competing financial interests or personal relationships that could have appeared to influence the work reported in this paper
